# Analysis of the Effects of *IL-6 -572 C/G*, *CRP -757 A/G*, and *CRP -717 T/C* Gene Polymorphisms; IL-6 Levels; and CRP Levels on Chronic Periodontitis in Coronary Artery Disease in Indonesia

**DOI:** 10.3390/genes14051073

**Published:** 2023-05-12

**Authors:** Sanggap Indra Sitompul, Budi Susetyo Pikir, Aryati, Citrawati Dyah Kencono Wungu, Shafira Kurnia Supandi, Monika Estherlita Sinta

**Affiliations:** 1 Doctoral Program of Medical Science, Faculty of Medicine, Universitas Airlangga, Surabaya 60132, Indonesia; sanggap.in.sitompul-2020@fk.unair.ac.id; 2 Medical Staff Group of Cardiology, Doris Sylvanus Hospital, Palangka Raya 73111, Indonesia; 3 Department of Cardiology and Vascular Medicine, Faculty of Medicine, Universitas Airlangga, Surabaya 60132, Indonesia; 4 Department of Clinical Pathology, Faculty of Medicine, Universitas Airlangga, Surabaya 60132, Indonesia; 5 Department of Physiology and Medical Biochemistry, Faculty of Medicine, Universitas Airlangga, Surabaya 60132, Indonesia; 6 Department of Periodontics, Faculty of Dental Medicine, Universitas Airlangga, Surabaya 60132, Indonesia; 7 Medical Staff Group of Oral Health, Doris Sylvanus Hospital, Palangka Raya 73111, Indonesia

**Keywords:** chronic periodontitis, c-reactive protein, coronary artery disease, gene polymorphism, interleukin-6

## Abstract

Interleukin 6 (IL-6) and C-Reactive Protein (CRP) play an important role in chronic periodontitis with coronary artery disease (CAD). Genetic factors can affect a person’s risk of CAD, which affects one-third of the population. This study investigated the role of *IL-6 -572 C/G*, *CRP -757 A/G*, and *CRP -717 T/C* gene polymorphisms. IL-6 and CRP levels on the severity of periodontitis in CAD in Indonesia were also evaluated. This case-control study was conducted with mild and moderate–severe chronic periodontitis groups. A path analysis test was conducted with Smart PLS with a 95% confidence interval to determine the significant variable for chronic periodontitis. Our study revealed that the effects of *IL-6 -572 C/G*, *CRP -757 A/G*, and *CRP -717 T/C* gene polymorphisms on IL-6 levels and CRP levels were not significant. IL-6 and CRP levels were not significantly different between the two groups. We found that IL-6 levels had a significant effect on CRP levels in periodontitis patients with CAD (path coefficient 0.322, *p* = 0.003). *IL-6 -572 C/G*, *CRP -757 A/G*, and *CRP -717 T/C* gene polymorphisms had no effect on the severity of chronic periodontitis in CAD patients in the Indonesian population. We also observed no apparent effects of the influence of gene polymorphisms in *IL-6 -572 C/G*, *CRP -757 A/G*, and *CRP -717 T/C* genes. Although the IL-6 and CRP levels were not significantly different between the two groups, IL-6 levels affected CRP levels in periodontitis patients with CAD.

## 1. Introduction

Coronary artery disease (CAD) is a cardiovascular disorder due to atherosclerosis or atherosclerotic occlusion of the coronary arteries [1]. It is estimated that 17.9 million people died from cardiovascular disease, heart attacks, and strokes in 2019 [2]. Several risk factors contribute to the development of coronary atherosclerosis, including diabetes mellitus, hypertension, smoking, a diet high in saturated fat, a lack of exercise, inflammatory factors [3], endothelial progenitor cells (EPCs) [4], and chronic periodontitis [5].

Periodontitis is defined as a chronic infectious disease of supporting tissues of teeth. It is associated with gene variations and environmental interaction [6,7]. This disease is a health problem, especially in developing countries [8]. The prevalence and severity of periodontitis increase with age, and this disease occurs frequently in males [9,10]. Moreover, periodontitis is associated with CAD [11,12] and an increase in early mortality [8].

The relationship between periodontitis and cardiovascular disease can develop through direct and indirect pathways. The direct pathways include bacterial invasion and infection, and the indirect pathways include increases in C-Reactive protein (CRP) and interleukin-6 (IL-6) [13,14]. Several studies reported that the involvement of CRP and CAD is associated with periodontitis [15,16,17,18]. Additionally, inflammatory mediators are also related to periodontitis [19,20].

Polymorphisms in the promoter region of the IL-6 gene trigger variations in the transcription [21]. The most frequent polymorphisms occur in *572 C/G* and *174 G/C* genes. The two SNPs influencing IL-6 expression and serum levels are associated with periodontitis susceptibility; however, their geographic and ethnic distribution is distinct [22,23]. The associations between IL-6 polymorphisms and periodontitis have been extensively studied. *IL-6 -572* may be a genetic risk factor for periodontitis patients in Asian populations, especially the Chinese population [24]. The *IL-6 -174* polymorphism is rare in the Chinese population [25]. Several studies have also shown a relationship between IL-6 polymorphisms and CAD, especially *IL-6 -572 C/G* [26,27,28]. Furthermore, a study reported the polymorphism of *IL-6 -572 G/C* is correlated with plasma concentrations of IL-6 [29].

CRP gene polymorphisms affect CRP levels in serum. Moreover, about 35–40% of variations in CRP levels between individuals are inherited [19]. Polymorphisms are associated with increased plasma CRP levels, *-717 A/G*, and *-757 T/C* genes [30]. A significant relationship has been reported between *CRP -757 T/C* and chronic periodontitis [31]. They play an important role in the pathophysiology of carotid atherosclerosis [32]. Meanwhile, the *CRP -717* polymorphisms have been shown to be associated with CAD [33] and periodontal health in Indonesia [34]. Studies about the relationship between IL-6 polymorphisms, CRP with chronic periodontitis, and CAD are limited, including studies on polymorphisms of *IL-6-572C/G* and CRP+1444 C/T [25,35].

To our knowledge, there are no studies on *IL-6 -572 C/G*, *CRP -757 A/G*, and *CRP -717 T/C* gene polymorphisms; IL-6 levels; and CRP levels in chronic periodontitis related to CAD patients in Indonesia. Therefore, we investigated the roles of *IL-6 -572 C/G*, *CRP -757 A/G*, and *CRP -717 T/C* gene polymorphisms; IL-6 levels; and CRP levels on the occurrence of chronic periodontitis related to CAD patients.

## 2. Materials and Methods

### 2.1. Study Design

This study was an analytic observational study with a case-control study. The. inclusion criteria were CAD patients with at least 20 teeth who underwent coronary angiography from December 2021 to October 2022 at dr. Doris Sylvanus Palangka Raya, Indonesia, and did not undergo six months of periodontal treatment. CAD patients with moderate–severe periodontitis were used as a case group, and patients with mild chronic periodontitis were used as a control group. The exclusion criteria were not using antibiotics and anti-inflammatories in the last three months, acute myocardial infarction, pregnancy, fever, pneumonia including COVID-19, and kidney failure. The sample size of each group was 40, and the samples were obtained by consecutive sampling.

Chronic periodontitis was diagnosed according to the assessment of the probing depth in 6 index teeth (16, 21, 24, 36, 41, 44) by measuring the depth of the buccal, mesial, lingual, and distal areas [36] with the Hu-Friedy PCPUNC instrument and conducting panoramic photo examination to determine alveolar reabsorption. A mean probing depth limit of 4 mm if ≥4 mm was included in the case group (moderate-severe periodontitis), while <4 mm was included in the control group (mild periodontitis). 

Stenosis was considered significant if at least 70% of the main coronary arteries were in one angiographic projection, at least 50% of two projections, and 50% of the main coronary arteries [37] using Quantitative Coronary Angiography (QCA) Allura FC. Interviews, physical examinations, and clinical laboratories were conducted to determine gender, age, dyslipidemia [38], consumption of lipid-lowering drugs, hypertension (systolic >140 and diastolic >90 mmHg measured twice/previous history/consumption of anti-hypertensives), smoking [39], obesity and diabetes mellitus (diagnosed by Internist/previous history/consumption anti-DM drugs). This study was approved by the Research Ethics at RSUD, dr. Doris Sylvanus Palangka Raya, Indonesia (Number: 5641/UM-TU/RSUD/11-2021). All subjects received and signed the informed consent form.

### 2.2. DNA Isolation

Blood samples were collected through the cubital vein. Samples were put into each vacutainer containing EDTA anticoagulant. Peripheral blood mononuclear cell (PBMC) isolation with the addition of Ficoll was performed using the Genomic DNA Mini Kit (Blood/Cultured Cell) (Cat. No. GB100, Geneaid Biotech Ltd., New Taipei City, Taiwan), according to the manufacturer’s recommended procedures. The results of DNA extraction were stored at −20 °C until further analysis. DNA quantification was measured using the NanoDrop TM One spectrophotometer (Thermo Fisher Scientific Inc., Wilmington, NC, USA).

### 2.3. Genotyping Polymorphism of IL-6 and CRP

The PCR mix consisted of 12.5 μL TaqMan^®^ GTXpress Master Mix 2X (Cat. No. 4403311, Lot: 01324621, Applied Biosystem, Foster City, CA, USA), 1.25 μL TaqMan^®^ SNP Genotyping Assay 20X, 1-20 ng DNA sample, and nuclease-free water to a final volume of 25 μL. Amplification was performed using a CFX 96 Touch™ Real-Time PCR (Bio-Rad, Hercules, CA, USA) with a setting of 20 s of enzyme activation at 95 °C, followed by 40 cycles of denaturation for 15 s at 95 °C and annealing extension for 1 min at 60 °C. Sample genotype was measured based on each allele’s Relative Fluorescence Unit (RFU). Materials for real-time PCR were TaqMan^®^ Pre-Designed SNP Genotyping Assay Reagent, Thermo Fisher Scientific Baltics UAB V. Graiciuno 8 LT-02241 Vilnius Lithuania, cat No. 4351379, C32343415_10 (size S) (rs3093059), C318207_10 (size S) (rs2794521), and C__11326893_10 (size S) (rs1800796). QuantStudio 5 (Applied Biosystems, Waltham, MA, USA) was used as a thermocycler.

Forward Primer (with M13 tail) *IL-6 -572 C/G* gene polymorphism (rs1800796): 5′-GTAAAACGACGGCCAGTAGTGGGCTGAAGCAGGTGA-3′ (36-mer). Reverse Primer (with M13 tail): 5′-GCGGATAACAATTTCACACAGGCTTTGTTGGAGGGTGAGGG-3′ (41 -mer).

Forward Primer (with M13 tail) *CRP -757 A/G* gene polymorphism (rs3093059): 5′-GTAAAACGACGGCCAGTCCTTTGGAAAAGATGTATTCGG-3′ (39-mer). Reverse Primer (with M13 tail): 5′-GCGGATAACAATTTCACACAGGGACTCTACTACAAAGGATACGG-3′ (44-mer).

Forward Primer (with M13 tail) *CRP -717 T/C* gene polymorphism (rs2794521): 5′-GTAAAACGACGGCCAGTCCTTTGGAAAAGATGTATTCGG-3’ (39-mer. Reverse Primer (with M13 tail): 5′-GCGGATAACAATTTCACACAGGGACTCTACTACAAAGGATACGG-3′ (44-mer). DNA sequencing confirmation was conducted by 1st BASE DNA Sequencing Division, Apical Scientific Laboratory, Selangor, Malaysia.

### 2.4. IL-6 Levels

IL-6 levels in blood serum were measured by electrochemiluminescence immunoassay (ECLIA) analysis. The result was expressed with pg/mL units. Additionally, the result was determined by a calibration curve with a two-point calibration and a master curve provided through a reagent barcode, with normal values ≤7.00 pg/mL (Ref. 05109442 190, Roche Diagnostics GMBH Sandhofer Str 116 -68305 Mannheim).

### 2.5. CRP Levels

CRP levels were determined based on quantitative HsCRP in serum with the latex immunoassay principle. Agglutination was detected as a change in absorbance at a wavelength of 572 nm with the turbidimetric or immunoturbidimetric method and normal values ≤10 ng/mL (Ref. 6K26-30, Sentinel CH SpA Via Robert Koch 2 Milan 20152 Italy).

### 2.6. Data Analysis

Data were analyzed using SPSS statistics software for Windows, version 26, IBM Corp, Armonk, NY, USA. A chi-square test was used to determine the relationship between polymorphisms and chronic periodontitis. Continuous data were presented as mean ± standard deviation (SD) while dichotomous with frequency and percentage. Continuous data were tested for normality distribution with the Shapiro–Wilk test. An ANOVA test was performed if the distribution was normal, while Kruskal–Wallis test was used if data were not normally distributed. The relationship between *IL-6 -572 C/G*, *CRP -757 A/G*, and *CRP -717 T/C* gene polymorphisms; IL-6 levels; and CRP levels was determined. Genotype and allele frequencies were compared by Hardy–Weinberg equilibrium. *t*-test was used if data distribution was normal. The Mann–Whitney test was used if data were not normally distributed. The path analysis test was conducted with Smart PLS 3.04 GmbH, Gewerbering, Oststeibek, Germany, to determine the dominant variable for chronic periodontitis. A 95% confidence interval was used and was considered a significant difference if *p* ≤ 0.05.

## 3. Results

### 3.1. Subject Characteristics

Our subjects were predominantly male (72.5%) patients who were over <60 years (83.8%) and had hypertension (78.8%). Our study showed that they did not suffer from diabetes mellitus (DM) (70%), and the statuses of dyslipidemia, smoking or non-smoking, and obesity were almost the same in both groups. We found the *IL-6 -572 C/G* homozygote minor GG 5 gene polymorphism (6.3%), the *CRP -717 T/C* homozygote *CC 5* gene (6.3%), and the *CRP -757 A/G* minor homozygote GG 2 gene (2.5%), as shown in Table 1. The frequencies of the C and G alleles and the A and G alleles appeared similar to the frequencies in Asian populations (https://www.ncbi.nlm.nih.gov/snp/rs1800796, accessed on 23 February 2023) (https://www.ncbi.nlm.nih.gov /snp/rs3093059, accessed on 23 February 2023). The frequency of the C allele was higher than the T allele compared to Asian populations (https://www.ncbi.nlm.nih.gov/snp/rs2794521, accessed on 23 February 2023).

The frequency distributions of *IL-6 -572 C/G*, *CRP -757 A/G*, and *CRP -717 T/C* gene polymorphisms were 0.540, 0.764; 0.099, 0.144; 0.196, 0.907 (x^2^, *p*-value, respectively), according to the Hardy–Weinberg equilibrium. We observed no significant difference between the mild and moderate–severe chronic periodontitis groups. Therefore, the two groups were considered homogeneous (*p* > 0.05).

### 3.2. Polymorphism Analysis of the IL-6 -572 C/G Gene, the CRP -757 A/G Gene, and the CRP -717 T/C Gene for the Severity of Periodontitis, IL-6 Levels, and CRP Levels

Table 2 shows the percentage of genotypes and alleles of the *IL-6 -572 C/G* gene polymorphism in patients with moderate–severe periodontitis and mild periodontitis with CAD. The genotypes and alleles of the two study groups were not statistically different (*p* = 0.233).

Regarding the *IL-6 -572 C/G* gene polymorphism, the percentage of the GG genotype was higher in the moderate–severe periodontitis group (10%) than in the mild periodontitis group (2.5%). Our study demonstrated that 5% of the GG genotype was found in the moderate–severe periodontitis group, while none of the GG genotype was found in the mild periodontitis group based on the *CRP -757 A/G* gene. We also found that the percentage of the CC genotype was lower in the moderate–severe periodontitis group (5%) than in the mild periodontitis group (7.5%) based on the *CRP -717 T/C* gene. Genotypes CC, CG, and GG and alleles C and G in the *IL-6 -572 C/G* gene; genotypes AA, AG, and GG and alleles A and G in the *CRP -757 A/G* gene; and genotypes TT, TC, and CC and alleles T and C in the *CRP -717 T/C* gene were not significantly different between the two groups (*p* > 0.05).

Table 3 shows the polymorphism of the *IL-6 -572 C/G* gene. We found that the average IL-6 level was higher in the GG genotype than in the CC and CG genotypes; however, it was not statistically significant (*p* = 0.579). The *CRP -757 A/G* gene polymorphism showed a lower average HsCRP level in the GG genotype compared to the AA and AG genotypes. However, it was not significantly different (*p* = 0.842). The average HsCRP level was lower in the CC genotype (4.580 mg/L) compared to the TT and TC genotypes (8.002 mg/L; 9.842 mg/L); however, there was no apparent significant difference (*p* = 0.490).

### 3.3. Correlation between IL-6 Levels, CRP Levels, and the Severity of Periodontitis in CAD

**Figure 1:**
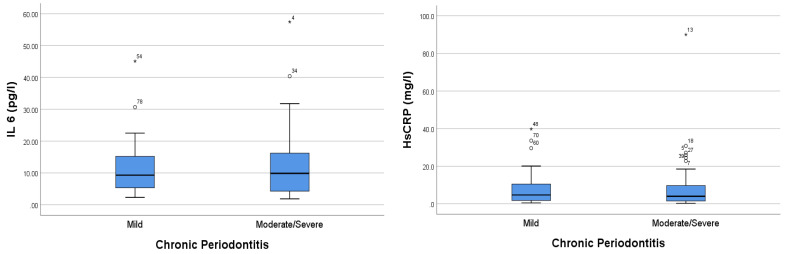
Comparison analysis of IL-6 and HsCRP levels in periodontitis patients with CAD. The * sign indicates the sample data number, which is an extreme outlier.

IL-6 and HsCRP levels were higher in the moderate–severe chronic periodontitis group than in the mild periodontitis group. Our results showed that the IL-6 levels in the mild and moderate–severe periodontitis groups were 11.245 ± SD 8.646 and 12.982 ± 11.752, respectively. Moreover, the CRP levels in the mild periodontitis and moderate–severe periodontitis groups were 7.675 ± SD 9.143 and 9.420 ± 15.548, respectively (Figure 1).

### 3.4. Path Analysis Effects of IL-6 -572 C/G, CRP -757 A/G, and CRP -717 T/C Gene Polymorphisms; IL-6 Levels; and CRP Levels on Chronic Periodontitis in CAD

**Figure 2:**
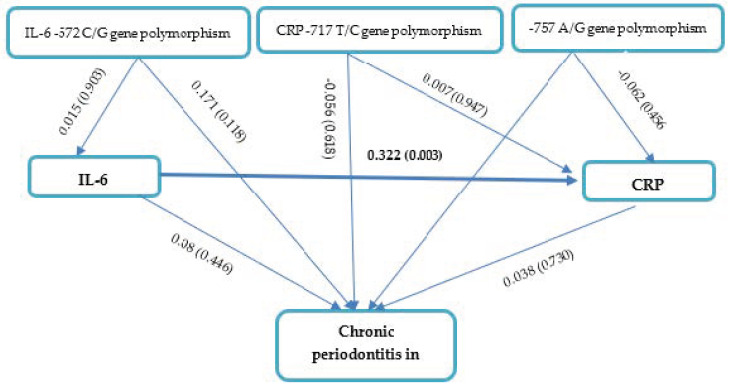
Path Analysis Effects of *IL-6 -572 C/G*, *CRP -757 A/G*, and *CRP -717 T/C* gene polymorphisms; IL-6 levels; and CRP levels on chronic periodontitis in CAD.

*IL-6 -572 C/G*, *CRP -757 A/G*, and *CRP -717 T/C* gene polymorphisms; IL-6 levels; and CRP levels had no apparent effects on the severity of chronic periodontitis in CAD based on the path analysis (Figure 2 and Table 4). Our results also demonstrated that *CRP -757 A/G*, *CRP-717 T/C*, and *IL-6 -572 C/G* gene polymorphisms had no effect on IL-6 levels (*p* > 0.05). Interestingly, IL-6 levels affected CRP levels in periodontitis patients with CAD (path coefficient 0.322, *p* = 0.003).

## 4. Discussion

### 4.1. Gene Polymorphisms in IL-6 -572 C/G, CRP -757 A/G, and CRP -717 T/C

The present study found no association between *IL-6-572 C⁄G* gene polymorphism and severe chronic periodontitis-related CAD in Indonesia. The mild and. moderate–severe periodontitis groups showed no significant differences in the. proportions of genotypes CC, CG, and GG or alleles C and G. We also found that IL-6 levels were the highest in the GG genotype compared to AA and AG; however, this was not statistically significant.

The etiology and pathogenesis of periodontal disease are complex [40]. The individual risk of periodontitis can be influenced by genetic factors [41], and the disease affects one-third of the population [42]. The study focuses on the effects of *IL-6 -572 C/G* gene polymorphisms on chronic periodontitis in CAD, which remain unclear. Based on the previous study, the *IL-6 -572 C ⁄G* gene polymorphism did not correlate with chronic periodontitis susceptibility, but it was significantly different between the CAD and non-CAD groups [25].

Polymorphisms have been reported to be related with periodontitis. These genetic risk factors play an important role in Asian populations, especially the Chinese population [24], and in Europeans [43]. In contrast, polymorphism *IL-6 -572* is not associated with periodontitis in the Iranian population [25]. Polymorphisms are associated with a susceptibility and risk factors of CAD [44]. Additionally, it is also associated with family histories of CAD [45]. A recent study showed a relationship between the *IL-6 -572 C/G* polymorphism and CAD. The CG and GG genotypes increase the risk of periodontitis-related CAD [25]. Gene polymorphisms in IL-6 influence IL-6 expression and cause. variations in transcription and expression between individuals [46,47]. The increase in IL-6 levels can be influenced by the *IL-6 -572* gene polymorphism [29], idiopathic pulmonary arterial hypertension [48], DVT [49], and the interaction of the IL-6 gene promoter haplotype [45].

No studies have focused on the *CRP -757 A/G* and *CRP -717 T/C* gene polymorphisms for chronic periodontitis in CAD. Our study showed no significant difference in the. proportions of the AA, AG, and GG genotypes or the A and G alleles (*-757 A/G*). We did not observe significant differences in the proportions of the genotypes TT, TC, CC, T, and C (*-717 T/C*) and the CRP levels in both groups. The highest mean CRP levels were in the AA genotype in the *CRP -757* gene and the TC genotype in the *CRP -717* gene, but they were not statistically significant. Similar studies also reported a significant association of CRP *-757 T/C* with chronic periodontitis in South India and high-risk TT genotypes [30]. The C allele is associated with increased CRP levels [33,50]. However, other studies have found an interaction effect between gender and obesity on increasing HsCRP [51]. This condition reinforces the genetic influence on CRP production [52].

The *CRP-717 T/C* gene is not related to periodontitis [31,53] including in the Indonesian population [54]. It provides a complementary indicator of periodontitis risk [33]. Another study reported that the *CRP -717* polymorphism predisposes to CAD allele A [32]. A relationship was also reported between CRP gene polymorphisms and increased CRP lebels in several populations, including -717 and -757 [29]. Our study reveals no effects of polymorphism on the severity of periodontitis in CAD patients. The results might have been affected by the sample size, the interaction of environmental and confounding factors [55], the differences in allele and genotype frequencies, and the heterogeneity of genetic susceptibility in several ethnic groups and races [49,56].

In the present study, the frequency of the G allele at *IL-6 -572* was lower than in the Asian population. In contrast, its frequency in the European population reached 95% [57]. Allele G at *CRP -757* showed a frequency of 14.4%, which is lower than the frequencies in Asia (16%) and the European population (6%) [58]. The frequency of the C allele in *CRP -717* is 26.9% higher than in the Asian population (16%) and in Europe (28%) [59]. A study reported that *-717 C/T* dominant C allele with homozygote recessive in Iranian population with chronic periodontitis [60]. Periodontitis was found in -717 dominant genotypes AA (46.1%), AG (43%), GG (10.7%), 757 TT (81.5%) TC (16.9%), and CC (1.5%) in the South Indian population [25]. The IL-6 and CRP gene polymorphisms might be influenced by IL-6 and CRP levels, especially in terms of the sample size, the limited number of polymorphism loci, and the multifactorial influences regulating these inflammatory mediators.

### 4.2. IL-6 and CRP Levels

Our study emphasizes the previous study concerning the significant effect of IL-6 on increasing CRP levels in chronic periodontitis with CAD. IL-6 is an inflammatory mediator encoded by the *IL-6* gene. It is involved in immunological, infection, inflammatory mechanisms, and polymorphisms in the *IL-6* gene region that affect physiological function and other cytokine imbalances [61,62].

Periodontitis is associated with increased IL-6 [16] and CRP levels [17,63,64]. A relationships between IL-6 and CRP were extensively studied. CRP is an inflammatory marker and is synthesized in the liver. CRP can trigger several cytokines, such as IL-6, IL-1, TNF-α, and IFN-α [65]. Furthermore, previous studies reported a relationship between IL-6 levels and increased CRP levels in the early stages of inflammation. IL-6 is synthesized and induces CRP [66,67].

Increased CRP levels in periodontitis have been reported [68,69,70]. Subgingival biofilm bacteria increase the total leukocyte count, including CRP, which is closely related to the patient’s systemic status [71]. IL-6 induces vascular endothelial growth factor (VEGF), matrix-metalloproteinase-1 (MMP-1), cathepsin L production [72], osteoclast differentiation, and bone resorption [73,74]. These conditions trigger the loss of the periodontal ligament and alveolar bone [41]. Furthermore, RANKL or OPG has a potential marker of alveolar bone damage [75,76,77].

IL-6 and CRP play important roles in forming CAD plaques [17,26,78,79,80]. CRP is a major parameter for treating CAD patients with periodontitis [17,81,82]. In contrast, CRP is not associated with periodontitis and CAD risk [49,58,83,84]. Another study showed the presence of CRP and IL-6 in periodontitis patients with CAD [85].

The results of the current study showed that the CAD with moderate–severe periodontitis group presented a higher level of IL-6 and CRP in comparison with the mild periodontitis group; however, our results were not statistically significant. This difference could be due to differences in examination methods, especially the probing depth limit [86], the involvement of Acute Myocardial Infarction [87], and the use of statins in CHD patients to reduce levels of inflammatory mediators [88]. In addition, IL-6 and CRP can be influenced by psychosocial or environmental pressure [89].

This study is the first study to analyze the effects of *IL-6 -572 C/G*, *CRP -757 A/G*, and *CRP -717 T/C* gene polymorphisms; IL-6 levels; and CRP levels on the severity of periodontitis in CAD, especially in the Indonesian population. However, there are several limitations. First, the sample size was only obtained from one center. Second, the type of polymorphisms studied were limited to the *IL-6 -572 C/G*, *CRP -757 A/G*, and *CRP -717 T/C* genes. Third, our study focused on the severity of chronic periodontitis in CAD and did not involve a normal group (without periodontitis). Further studies are needed to involve healthy controls and more centers.

## 5. Conclusions

The chronic periodontitis population with CAD had the majority of CC, AA, and TT genotypes in *IL-6 -572 C/G*, *CRP -757 A/G*, and *CRP -717 T/C* C/G gene polymorphisms. Polymorphisms were not affected by the severity of chronic periodontitis, IL-6 levels, and CRP levels. In addition, IL-6 levels affected CRP levels in moderate–severe periodontitis patients with CAD. Further studies are warranted to involve IL-6 and CRP polymorphisms at other loci and with larger samples.

## Figures and Tables

**Table 1:** Subject characteristics.

Parameter		N	Percentage
Gender	Male	58	72.5
	Female	22	27.5
Age	<60 years	67	83.8
	≥60 years	13	16.3
Hypertension	No	17	21.3
	Yes	63	78.8
DM	No	56	70
	Yes	24	30
Smoking	No	42	52.5
	Yes	38	47.5
Dysplidemia	No	38	47.5
	Yes	42	52.5
Obesity	BMI < 25 kg/m^2^	44	55
	BMI ≥ 25 kg/m^2^	36	45
Periodontitis	Mild	40	50
	Moderate–severe	40	50
Gene polymorphism in *IL-6 572 C/G*	CC	40	50
	CG	35	43.8
	GG	5	6.3
	Alelles		
	C	116	72.5
	G	44	27.5
Gene polymorphism in *CRP 717 T/C*	TT	42	52.5
	TC	33	41.3
	CC	5	6.3
	Alelles		
	T	117	73.1
	C	43	26.9
Gene polymorphism in *CRP 757 A/G*	AA	59	73.8
	AG	19	23.8
	GG	2	2.5
	Alelles		
	A	137	85.6
	G	23	14.4
IL-6 levels (pg/L)	Range	1.88–57.47	
	Mean ± SD	12.11 ± 10.29	
CRP levels (mg/L)	Range	0.3–90	
	Mean ± SD	8.55 ± 12.70	
Mean PD	Range	2.54–6.33	
	Mean ± SD	4.02 ± 0.62	

**Table 2:** Relationship between polymorphisms and alleles of the *IL-6 -572 C/G*, *CRP -757 A/G*, and *CRP -717 T/C* genes with chronic periodontitis.

	Chronic Periodontitis				∑	*p*
	Mild		Moderate–Severe			
	N	%	N	%		
Gene polymorphism in *IL-6 -572 C/G*						
Genotype						0.233
CC	23	57.5	17	42.5	40	
CG	16	40	19	47.5		
GG	1	2.5	4	10	35	
					5	
Total	40	100	40	100	80	
Alleles						0.111
C	63	78.8	53	66.2	11	
G	17	21.2	27	33.8	6	
					44	
Total	80	100	80	100	16	
					0	
Gene polymorphism in *CRP -757 A/G*						
Genotype						0.332
AA	31	77.5	28	70	59	
AG	9	22.5	10	25	19	
GG	0	0	2	5	2	
Total	40	100	40	100	80	
Alleles						0.368
A	71	88.8	66	82.5	13	
G	9	11.2	14	17.5	7	
					23	
Total	80	100	80	100	16	
					0	
Gene polymorphism in *CRP -717 T/C*						
Genotype						0.653
TT	19	47.5	23	57.5	42	
TC	18	45	15	37.5	33	
CC	3	7.5	2	5	5	
Total	40	100	40	100	80	
Alleles						0.476
T	56	70	61	76.2	11	
C	24	30	19	23.8	7	
					43	
Total	80	100	80	100	16	
					0	

**Table 3:** Relationship between *IL-6 -572 C/G*, *CRP -757 A/G*, and *CRP -717 T/C* gene polymorphisms; IL-6 levels; and CRP levels in periodontitis and CAD.

Genotype	N	Mean	Min-Max	Standard Deviation	*p*-Value
IL-6 gene polymorphism -*572 C/G* on IL-6 levels (pg/L)					
CC	40	12.448	(1.88–45.13)	9.419	0.579
CG	35	11.131	(2.33–57.47)	10.869	
GG	5	16.314	(3.91–31.80)	13.768	
CRP gene polymorphism *-757 A/G* on CRP levels (mg/L)					
AA	59	9.231	0.3–90	13.986	0.842
AG	19	6.984	0.9–33.6	8.401	
GG	2	3.250	2.7–3.8	0.778	
CRP gene polymorphism *-717 T/C* on CRP levels (mg/L)					
TT	42	8.002	0.3–90	14.808	0.490
TC	33	9.842	0.5–39.8	10.482	
CC	5	4.580	0.5–11.4	4.906	

**Table 4:** Path Coefficients (Direct and Indirect effects).

	Direct		Indirect	
	Effect	*p*-Values	Effect	*p*-Values
Gene Polymorphism in *IL-6 -572* → Chronic periodontitis	0.171	0.118	0.001	0.940
Gene Polymorphism in *CRP -757* → Chronic periodontitis	0.115	0.292	−0.002	0.811
Gene Polymorphism in *CRP -717* → Chronic periodontitis	−0.056	0.618	0.000	0.984
Gene Polymorphism in *IL-6 -572* → IL-6	0.015	0.903		
Gene Polymorphism in *CRP -757*→ CRP	−0.062	0.456		
Gene Polymorphism in *CRP -717* → CRP	0.007	0.947		
IL-6 → CRP	0.322	0.003 *		
IL-6 → Chronic periodontitis	0.080	0.446	0.012	0.755
CRP → Chronic periodontitis	0.038	0.730		

* Significant (*p* < 0.05).

## Data Availability

All relevant data are within the paper.
